# Comparative Response of the Nigerian Indigenous and Broiler Chickens to a Field Caecal Isolate of* Eimeria* Oocysts

**DOI:** 10.1155/2017/2674078

**Published:** 2017-04-24

**Authors:** Lucas Atehmengo Ngongeh, Amaechi Onyeabor, Emeka Nzenwata, Gurama Kansalem Samson

**Affiliations:** Department of Veterinary Microbiology and Parasitology, College of Veterinary Medicine, Michael Okpara University of Agriculture, Umudike 7267, Abia State, Nigeria

## Abstract

Response of Nigerian indigenous (local) and broiler chickens to experimental* Eimeria* infections was investigated by measures of clinical signs, packed cell volume (PCV), body weights (BW), feed consumption, faecal oocyst counts (oocyst per gram), and microscopic intestinal lesions. Three-week-old chickens of each breed received single pulse infections with 2500, 5000, and 100.000 sporulated* Eimeria* oocysts. Infected birds were dull and passed bloody diarrhoea. OPG showed a dose related response but no significant difference between groups (*P* > 0.05). OPG was significantly higher in local chickens (*P* < 0.05) and varied significantly with time (*P* < 0.05). PCV declined significantly in infected birds within breeds and groups (*P* < 0.05); however, the decline in PCV was significantly greater in broilers (*P* < 0.05). Both breeds had significant BW gains (*P* < 0.05). BW gain varied between groups being significantly higher in the uninfected control broilers than in the infected broilers (*P* < 0.05). Comparatively, broilers gained significantly more BW than their local counterparts (*P* < 0.05). Feed intake increased significantly with time (*P* < 0.05) in both breeds. The* Eimeria* isolate was pathogenic to both breeds of chicken although clinical signs and lesions were more severe in indigenous chickens suggesting the breed's more susceptibility.

## 1. Introduction

There is a tremendous expansion in the commercial poultry in Nigeria [1] as poultry is one of the fastest and affordable means of meeting the protein requirements for adequate nutrition of the human population. Unfortunately, poultry diseases are a major threat to the poultry industry in the world [2]. Among the diseases affecting the poultry industry is coccidiosis [3]. Coccidiosis has been ranked as one of the most economically important diseases of domestic poultry due partially to the high morbidity and mortality it causes and the huge cost involved in its control [4, 5]. Coccidiosis is an intestinal parasitic disease caused by members of the intracellular parasite known as* Eimeria* [4, 6]. There are seven species of* Eimeria* species causing coccidiosis in chickens, of which* E. tenella, E. maxima, E. necatrix,* and* E. brunetti* are highly pathogenic whereas others like* E. acervulina, E. mitis,* and* E. praecox* are less virulent [4, 6]. Species of the genus* Eimeria* are predominantly host-specific; each species occurs in a single host species or a group of closely related hosts [7]. It usually affects chickens of 3 to 18 weeks, but it is more severe in chickens from 3 to 6 weeks [8]. The disease has been reported in many parts of Nigeria [9, 10] and has been established as a major parasitic disease of poultry [2, 11, 12]. The use of antimicrobial growth promoters is being reduced because of concern for the spread of antimicrobial resistance. One of the negative consequences associated with the prohibition of antibiotic growth promoters in commercial poultry production is the increase in intestinal infectious diseases, such as necrotic enteritis, coccidiosis, and cryptosporidiosis [13]. Coccidiosis affects both exotic (broilers, layers, and cockerels) and Nigerian indigenous chickens. Among the factors that influence host response to parasitic infections is breed of the host. Some breeds of animals show considerably more resistance than others and may survive exposure to infections which prove lethal to other breeds [14]. Evidence that domestic animals show variability in their ability to resist infections have been documented both within and between breeds of animals [14], and this was shown to have a genetic basis [15]. An enhanced capacity to resist infection is inherited and can be passed from parent to offspring usually as a dominant trait [16, 17].

Extensive research has been carried out regarding the immunological mechanism controlling intestinal cell mediated immune responses [6, 17–19]. The control of coccidiosis has been mainly by the use of anticoccidial drugs and vaccines [20, 21]. However, apart from the huge finances involved in these methods, the emergence of coccidiosis-resistant strains is decreasing the effectiveness of these approaches [21]. Finding genes associated with resistance to coccidiosis will be useful for efficient genetic selection for coccidiosis-resistant lines of chickens and in understanding the mechanisms underlying disease resistance in chickens [22, 23].

There is evidence that resistance and susceptibility to avian coccidiosis are associated with inheritance [24] and eventually chicken lines resistant to avian coccidiosis could be established by genetic selection [25]. No such documented evidence exists or is known to the authors about the Nigerian indigenous (local) chicken in comparison to their exotic counterparts such as the broilers and layers where some studies have already been conducted. However, like some indigenous breeds of animals such as the West African Dwarf (WAD) goat and Muturu and N'dama cattle that have been shown to be more resistant to helminth and trypanosome infections than their exotic counterparts [26–28], it is hoped that the Nigerian indigenous chicken will be more resistant to* Eimeria* infections than broilers and layers which are exotic. Such information if available can be exploited in the control of parasitic diseases of chickens particularly coccidiosis if one of the breeds of chickens is found to be more resistant to* Eimeria* infections than the other. In such a situation genes of the more resistant type of bird can be incorporated into the less resistant type through production of their hybrids, for example. There is need therefore to breach that dearth of information. Consequently, this study was conducted to compare the response of the Nigerian indigenous and broiler chickens to graded doses of a field caecal isolate of* Eimeria *oocysts as a preliminary step toward the identification of chicken breeds that can relatively resist coccidiosis and can therefore be reared as a sustainable control method for coccidiosis.

## 2. Materials and Methods

### 2.1. Experimental Birds and Management

The study was carried out at the Poultry Unit of the Teaching and Research Farm, College of Veterinary Medicine, Michael Okpara University of Agriculture, Umudike. Broiler chicks of Anak 2000 breed (commonly reared in Nigeria due to their big size and body conformation and white colour) were procured from a reputable source while Nigerian indigenous (normal feathered local chicken) chicks were obtained from local chicken breeders hatched from a parent stock from the Department of Animal Science, University of Nigeria, Nsukka, where they are maintained. The birds were acquired at the age of one day and brooded. All the birds were managed in deep litter system in the course of the experiment (after their first week of life on newspapers on the floor) and the litter was maintained dry by avoiding water spillage and by replacing litter with fresh dry wood shavings every two days. The birds were duly vaccinated against viral infections (Newcastle disease and infections bursal disease). Prophylactic treatment against* Eimeria* infections in the course of brooding was done with toltrazuril (Baycox 2.5% w/v oral solution) which was withdrawn before experimental infections. The birds were fed ad libitum throughout the brooding and experimental periods.

All relevant laws and codes of practice governing the experimental studies with life animals were complied as stipulated in [29], and the experimental protocol was approved by Michael Okpara University of Agriculture, Umudike Animal Ethics Committee.

### 2.2. Eimeria Isolate

Caeca of slaughtered broilers and layers were obtained from the slaughter house at Umuahia Urban Market. Some caeca were also collected from broiler chickens clinically sick of coccidiosis in Michael Okpara University of Agriculture, Umudike (MOUAU) poultry house. The caeca were incised and their contents washed out into large faecal cups and the coccidian oocysts recovered as described in [30]. Briefly the contents were washed through a sieve and the filtrate was allowed to sediment in the plastic faecal cups for 10 minutes. The supernatant was decanted and the sediment was placed in 50 mL Teflon tubes. The* Eimeria* oocysts in the sediment were recovered by adding saturated sodium chloride solution (NaCl). The tubes were allowed to stand for 10 minutes for the oocysts to float, and the tops were decanted into empty tubes and water was added to them to dilute the effect of the salt. The oocysts sedimented at the bottom of the tubes and were collected after decanting the supernatant. The oocysts were sporulated by introducing a 2.5% solution of potassium dichromate (KCr_2_07) and leaving it on the bench in the laboratory for 3 days in Petri dishes at room temperature (25°C). The sporulated oocysts were preserved in the refrigerator at 4°C until when they were used within two weeks of recovery.

### 2.3. Experimental Design and Infection

All chickens were screened for the presence of* Eimeria* oocysts two days before the experimental infection and were found to be free of natural infection. The broiler chickens were randomly distributed into four groups of eight birds each while the indigenous (local) chickens were randomly distributed into four groups of four birds each (only four birds per group due to their scarcity). Three groups of each breed of 3-week-old chickens were infected orally with varying doses of sporulated* Eimeria* oocysts by delivering the suspension into the mouth of each of the chickens using a tuberculin syringe which was rinsed with water each time following inoculation while the fourth group remained as uninfected controls (Table 1). Birds of each experimental group were placed in a separate pen. The course of the infection was monitored by measurement of feed consumption, body weight (BW), packed cell volume (PCV), clinical signs, faecal oocyst counts (OPG), and gross and microscopic lesions.

### 2.4. Feed Intake

Feed was weighed using a top loading balance (Camry Emperors, China) each time before introducing into the feeding trough and again weighed at the end of the day, and the difference was determined and recorded as the feed consumed each day for each group. The mean feed consumption per bird was determined by dividing the total amount of feed consumed per group by the number of birds in each group.

### 2.5. Body Weight

The birds were all weighed with a top loading balance (Camry Emperors, China) on day zero of the experiment at 21 days of age and subsequently every 4 days till the end of the study.

### 2.6. Packed Cell Volume

The PCV was taken at 4-day interval beginning from day zero of the experiment till the end of the study. Blood was collected via the jugular vein directly into heparinized capillary tubes (Camlab Ltd., Cambridge) for determination of PCV [31].

### 2.7. Clinical Signs

The birds were monitored closely for possible observable signs, namely, dullness, diarrhoea, clustering, drooping of wings, anorexia, and mortality throughout the course of the experiment starting on the second day after infection.

### 2.8. Faecal Oocysts Counts

Faecal samples were collected daily from each group of the experimental birds from the third day following inoculation and analyzed for the presence of oocysts using the floatation technique, but when more than 100 oocysts were present, the modified McMaster technique was adopted [32]. The first appearance of oocysts in any group was noted as the prepatent period. The oocyst counts were conducted every day from day 3 of the infection till patency was established and thereafter every 2 days till the end of the study.

### 2.9. Gross Lesion Score

At the end of the experiment, three birds (randomly picked) from each of the experimentally infected groups of both breeds were humanely sacrificed by cervical dislocation. On evisceration, presence of gross lesions especially haemorrhages and necrotic foci were looked for in the mucosa at various positions in the intestine (duodenum, jejunum, ileum, caeca, and rectum) following a longitudinal incision of the intestine. Coccidial lesion scoring was conducted for the caeca. Gross lesions were graded from 0 to 4 based on lesion score key [33].

### 2.10. Microscopic Lesions

Intestinal tissues from various sections of the intestine were collected for histopathology and detection of the developmental stages of* Eimeria* species. Histopathological studies were made in all sections of the intestine (duodenum, jejunum, ileum, caecum, and rectum) of three chickens randomly chosen from each of the groups at the end of the experiment (D25). Tissues were fixed in 10% formal saline solution. Following the fixation process, samples were dehydrated in alcohol, cleared in xylene, and then embedded in paraffin wax. The tissues were sectioned at 5 *μ*m and stained by haematoxylin and eosin (H&E). Stained tissues were mounted and examined under a light microscope.

### 2.11. Statistical Analysis

Data were analyzed using Univariate Analysis of Variance and probabilities of 0.05 or less were considered significant. All data of the faecal oocyst counts (OPG) were normalized using log_10_(OPG + 25).

## 3. Results

### 3.1. Clinical Signs

The infected birds exhibited the tendency to cluster together drooping their wings and were dull from days 3 to 7 and this was more obvious in the local chickens. The infected broilers passed brown to bloody diarrhoeic faeces for five days from day 10 while the indigenous chickens passed bloody mucoid diarrhoeic faeces from days 8 to 14.

**Figure 1:**
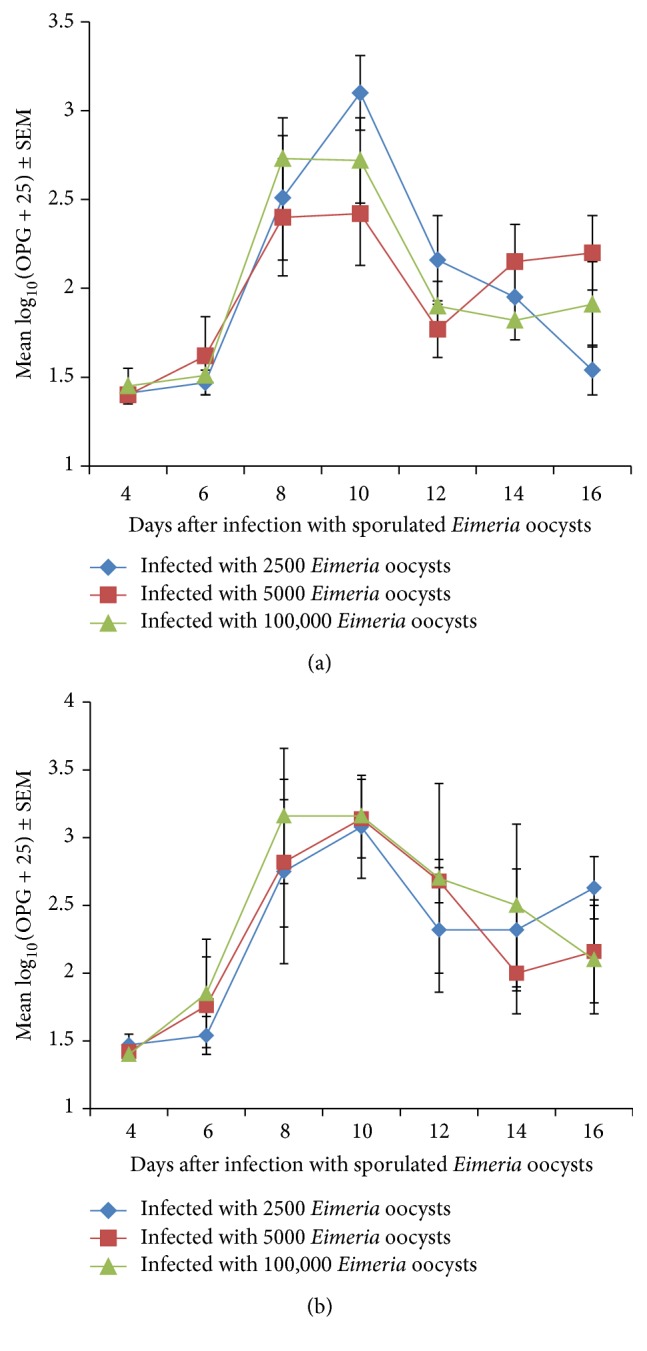
(a) Mean transformed OPG of broiler chicken infected with a caecal isolate of sporulated* Eimeria* oocysts. (b) Mean transformed OPG of Nigerian indigenous chicken infected with a caecal isolate of sporulated* Eimeria* oocysts.

Infections became patent on day 4 following infection in both broilers and Nigerian indigenous chickens infected with a caecal isolate of* Eimeria* sporulated oocysts shown by detection of oocysts in the faeces of the birds. OPG in the broiler groups increased gradually following patency and then rose sharply from day 6 to day 10 in 5000 and 100.000 dose level before leveling off while the 2500 dose level kept rising and peaked at day 10. The OPG started declining in all groups from day 10 and continued declining to the end of the study although there were mild fluctuations in 5000 and 100.000 dose levels (Figure 1(a)). On day 8 the OPG was higher in the 100.000 level than in other groups while it was highest in the 2500 dose level on day 10 (Figure 1(a)) but the difference between the groups was not significant (*P* > 0.05).

Following patency on day 4 in the Nigerian indigenous chickens the OPG rose sharply in all groups up to day 10 with the 100.000 dose level being the highest while the 5000 and 2500 dose levels were moderate and low, respectively (Figure 1(b)); however, there was no significant difference between groups (*P* > 0.05). The OPG of the Nigerian indigenous chickens was significantly higher than that of the broiler chickens (*P* < 0.05) as shown on Figures 1(a) and 1(b) (with mean OPG of 2.25 ± 0.7 and 1.9 ± .05 for Nigerian indigenous and broiler chickens, resp.). Generally the OPG varied significantly with time (*P* < 0.05).

### 3.2. Packed Cell Volume

**Figure 2:**
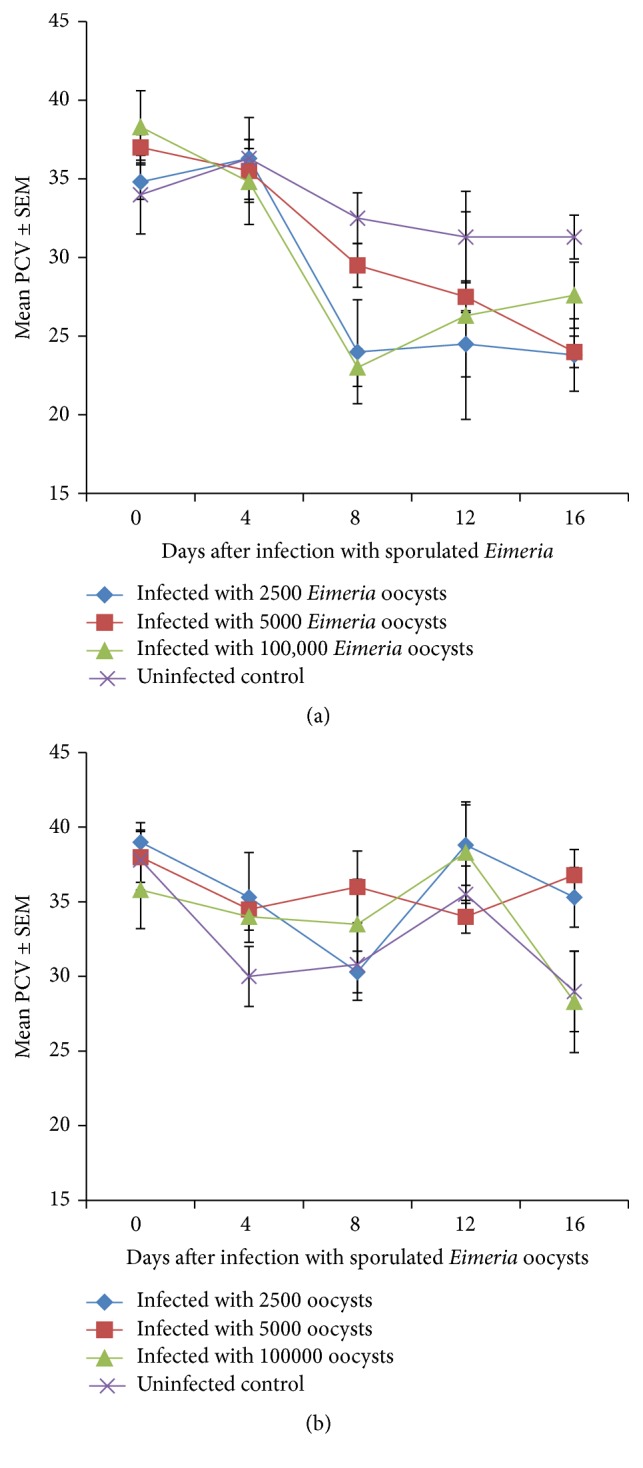
(a) Mean PVC of broiler chickens infected with a caecal isolate of sporulated* Eimeria* oocysts. (b) Mean PVC of Nigerian indigenous chickens infected with a caecal isolate of* Eimeria* sporulated oocysts.

The PCV of all experimental chickens declined from day 4 to day 8 and even up to day 16 in some of the groups; however, the decline was more severe in the infected chickens compared to the uninfected control chickens (Figure 2(a)). The decline in PCV with time within breeds was significant (*P* < 0.05). PCV also varied significantly between groups in a given breed being significantly lower in the infected groups than in the uninfected controls (*P* < 0.05). The decline in PCV in broilers was greatest in the 100.000, moderate in the 5000, and least in the 2500 dose levels (Figure 2(a)). In the Nigerian indigenous chickens the PCV generally fluctuated declining significantly (*P* < 0.05) from day 0 to day 4 or day 8 in some groups (Figure 2(b)). Generally, the decline in PCV was more severe in broilers than in the indigenous chickens and the difference was significant (*P* < 0.05) (Figures 2(a) and 2(b)).

### 3.3. Body Weight

**Figure 3:**
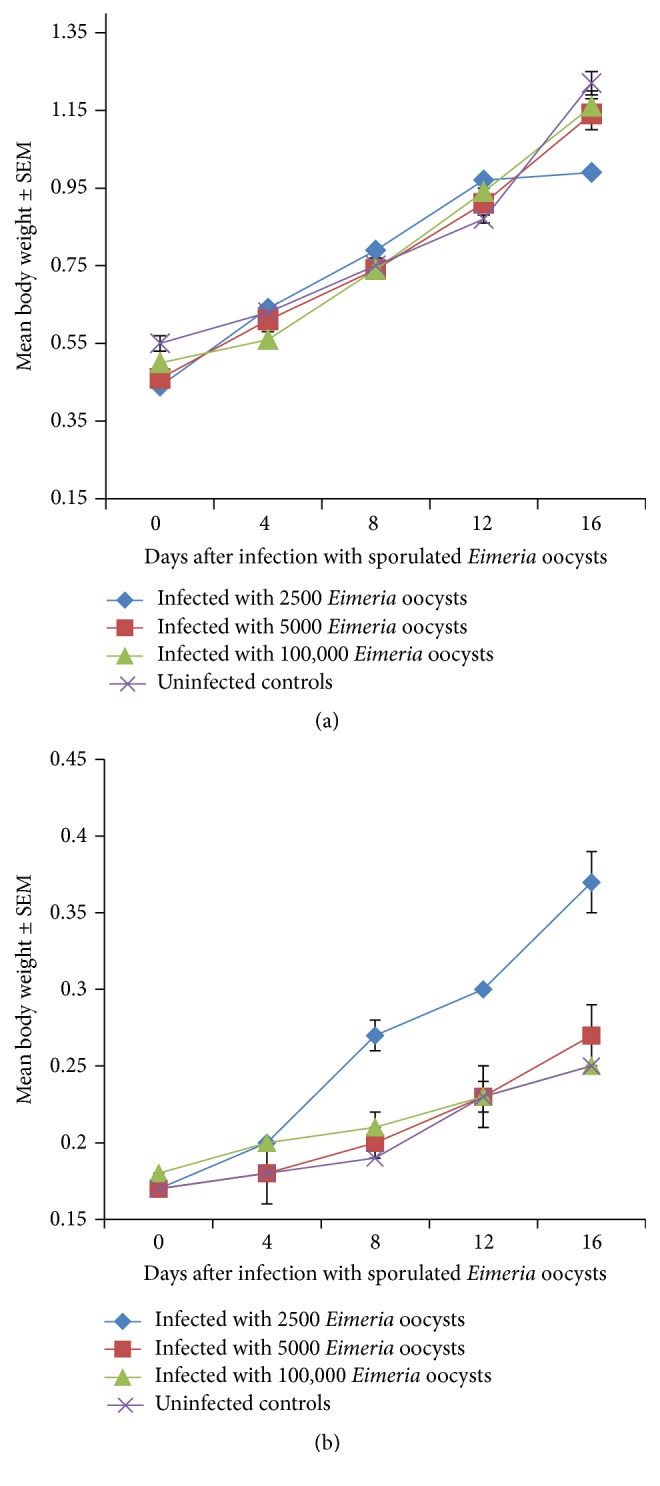
(a) Mean body weight of broilers infected with a caecal isolate of sporulated* Eimeria* oocysts. (b) Mean body weight of Nigerian indigenous chicken infected with a caecal isolate of sporulated* Eimeria* oocyst.

The body weight generally increased in all groups of chickens irrespective of their breed as the study progressed (Figures 3(a) and 3(b)) with resultant significant weight gains at the end of the study (*P* < 0.05). The body weight gain was significantly greater (*P* < 0.05) in the uninfected control broilers especially towards the end of the study from day 12 to day 16 (Figure 3(a)). In the indigenous chickens, however, the gain in body weight was surprisingly higher in the 2500 dose level group than other groups (Figure 3(b)). As expected, the rate of body weight gain was significantly higher (*P* < 0.05) in the broiler chickens than in their indigenous counterparts where rate of weight gain was very low (Figures 3(a) and 3(b)).

Both breeds of birds had significant weight gains (*P* < 0.05) by the end of the study irrespective of whether they were infected or not. Weight gain varied between groups being significantly higher in the uninfected control broilers than in the infected broiler groups (*P* < 0.05). Comparatively, broilers gained significantly more weight than their indigenous counterparts (*P* < 0.05).

### 3.4. Feed Consumption

**Figure 4:**
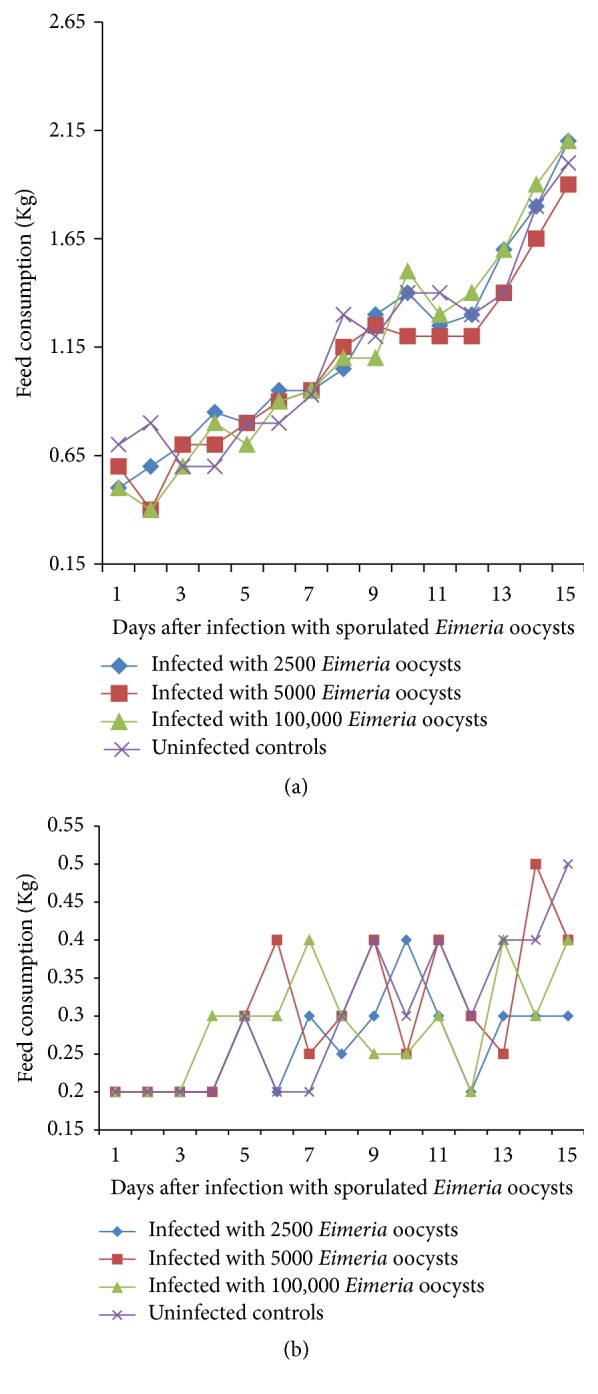
(a) Feed intake of broilers infected with a caecal isolate of sporulated* Eimeria* oocysts. (b) Feed intake of Nigerian indigenous chicken infected with a caecal isolate of sporulated* Eimeria* oocysts.

Feed consumption generally increased in all groups of broilers although with some mild fluctuations as the study progressed (Figure 4(a)). However, there were a lot of variations in the feed intake of the indigenous chickens although the feed consumption did generally increase with time (Figure 4(b)). The indigenous chickens infected with the 100.000 dose level had a lower feed intake than groups from day 9 to day 13 (Figure 4(b)). Feed intake increased significantly (*P* < 0.05) as the study progressed. The feed intake of broilers was significantly greater than that of the indigenous chicken (*P* < 0.05) (Figures 4(a) and 4(b)). For example, on day 15, the mean feed intake of broilers and indigenous chickens was 0.78 ± 0.01 and 0.25 ± 0.01 kg, respectively. Feed intake also varied between groups being significantly higher in the uninfected control birds than in the infected groups (*P* < 0.05).

### 3.5. Gross Lesions

Haemorrhages were common in the intestinal mucosa of infected chickens in both breeds of birds. Haemorrhages were most common and most prominent in the caeca in both breeds of birds and became more severe as the dose level increased. The gross lesion scores ranged from 0 to 2 in both breeds of chickens.

### 3.6. Histopathological Lesions

**Figure 5:**
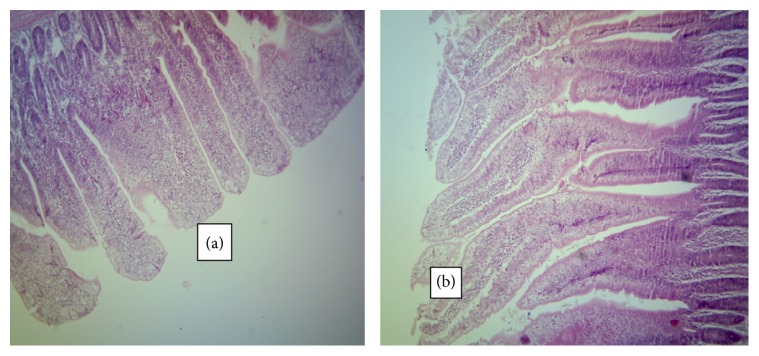
Caeca of an uninfected broiler (a) and an uninfected indigenous chicken (b) showing normal architecture of the mucosa (H&E ×100).

**Figure 6:**
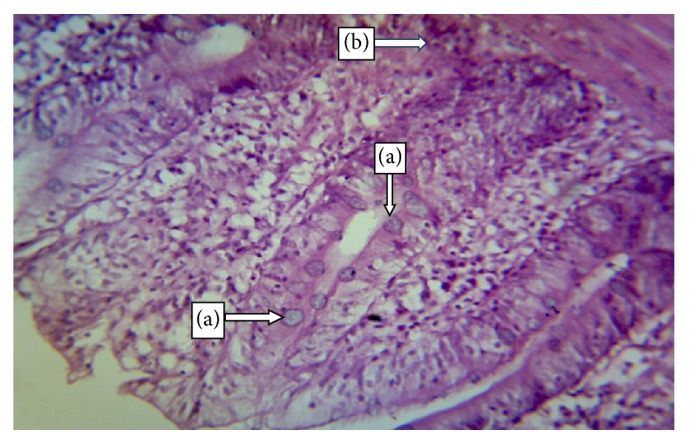
Caecum of a broiler infected with 100.000 sporulated* Eimeria* oocysts showing developmental stages of* Eimeria* in the epithelial mucosa (a) and haemorrhagic foci (b) (H&E ×400).

**Figure 7:**
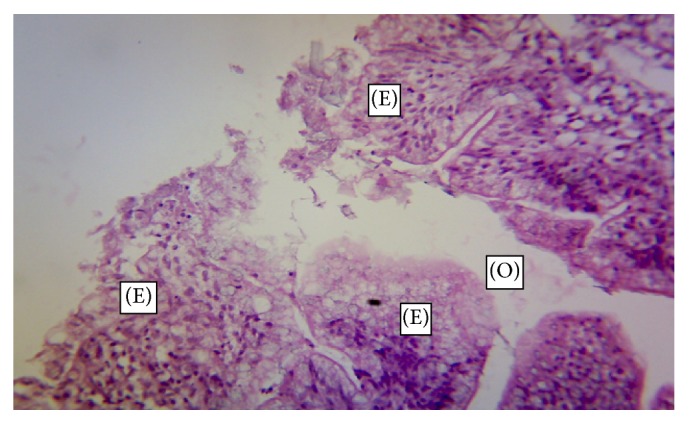
Caecum of a broiler infected with 5000 sporulated* Eimeria* oocysts showing erosion (E) of the epithelial lining of the mucosa and oedema (O) (H&E ×400).

**Figure 8:**
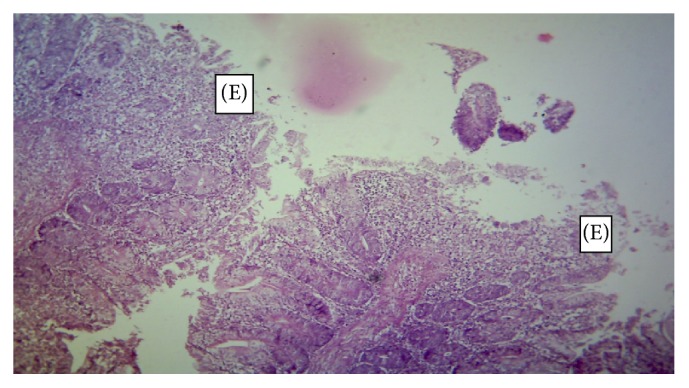
Caecum of a Nigerian indigenous chicken infected with 2500 sporulated* Eimeria* oocysts showing erosion (E) of the epithelial mucosa (H&E ×400)

**Figure 9:**
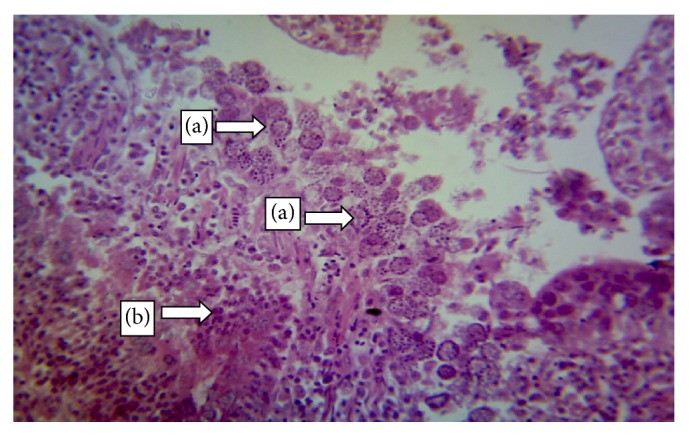
Duodenum of an indigenous chicken infected with 100.000 sporulated* Eimeria* oocysts showing developmental stages of* Eimeria* within the epithelium (a) with mononuclear cell infiltration (b) (H&E ×400).

**Figure 10:**
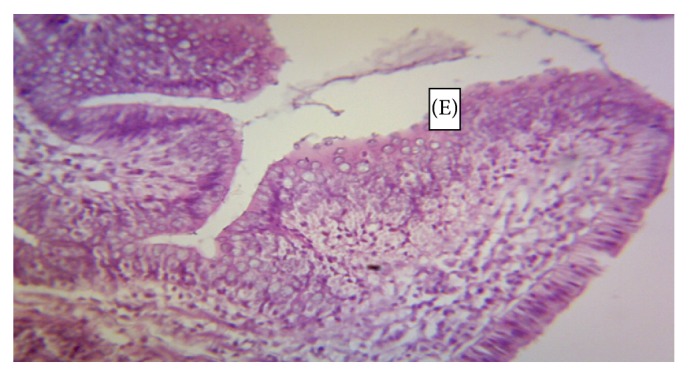
Duodenum of a broiler infected with 2500 sporulated* Eimeria* oocysts showing erosion (E) of the epithelial lining of the mucosa (H&E ×400).

**Figure 11:**
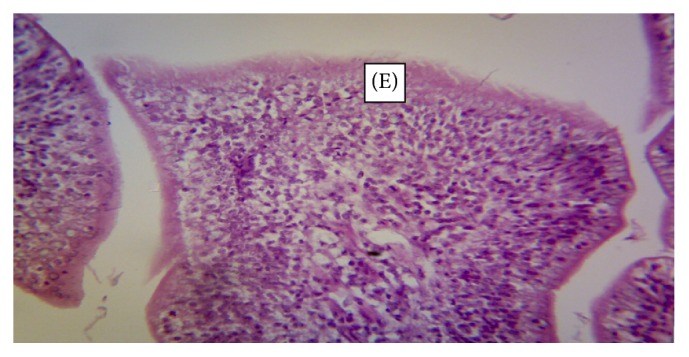
Ileum of a broiler infected with 2500 sporulated* Eimeria* oocysts showing minimal erosion (E) of the epithelial lining of the mucosa (H&E ×400).

The normal microscopic morphology of the caeca of an uninfected broiler (Figure 5(a)) and local (Figure 5(b)) chickens is shown. Microscopic changes were observed in the intestinal tissues of infected chickens and included changes in the morphology of the villi such as villous erosion, disruption of the architecture of the epithelial lining, epithelial desquamation and oedema (Figures 6–11), and mononuclear inflammatory cellular infiltration of the lamina propria and hyperplasia of the epithelial crypts (Figures 6–11). Developmental stages of the parasites (schizonts) were also observed (Figure 6). The histological findings reported here were common in both the broilers and indigenous chickens. The severity of lesions increased with increase in the dose level of the sporulated* Eimeria* oocysts.

## 4. Discussion

Both breeds of chickens were susceptible to the infection demonstrated by patency of the infections in the infected birds. There was a marked similarity between some of the clinical signs, gross lesion score, and microscopic lesions presented with those usually exhibited by chickens with clinical coccidiosis. Histology also revealed the presence of developmental stages of* Eimeria* species in the tissues of the intestine and these were common in all parts of the intestine suggesting that the caecal isolate of* Eimeria* used comprised various species of* Eimeria*. This concords with the findings of Ngongeh [30] who had observed that a caecal isolate from broilers and layers reared in Nsukka area of Enugu state in eastern Nigeria (the same area) was infective and caused both gross and microscopic lesions in different parts of the intestines of two-week-old broilers in experimental infections, and oocysts were recovered from all parts of the intestine.* Eimeria* infections usually occur as mixed infections in chickens in nature, a situation which is common in both intensive and free range system of poultry management [10, 34]. The occurrence of clinical signs and intestinal mucosal haemorrhages and erosions of the infected chickens is an indication that* Eimeria* species isolated from birds in some parts of Abia state were pathogenic to the birds.

The response of the chickens to the* Eimeria* infections was dose related because the clinical signs, gross lesion scores, and microscopic lesions became more severe as the dose level increased. It has been reported that the outcome of most parasitic infections is influenced by the size of the inoculum ingested or administered to the host [32, 35]. The result obtained in this study emphasizes the need to implement control measures that will keep the* Eimeria *oocysts load in the poultry houses to the barest minimum to avoid the ingestion of high quantities of the oocysts.

The fact that no mortality occurred in both broilers and indigenous chickens may be due to the stringent hygiene practices that were maintained to avoid reinfection. The pathology (gross and microscopic lesions) inflicted varied with increasing dose level with the lesions becoming more severe as the dose level of the* Eimeria* oocysts increased. The dose level-based kind of response was also seen in the OPG where the indigenous chickens infected with the highest dose level had a corresponding high OPG and most severe clinical signs. Also worthy of notice was the fact that the response of the chickens to the infections was influenced by the breed since the indigenous birds showed more severe and prolonged clinical signs such as dullness, droopy wings, and bloody diarrhoea, and at autopsy fresh haemorrhages with frank blood were still very common in the indigenous birds while most haemorrhages/ulcers in the broilers had advanced in healing evidenced by nodules with scabs.

The general decline and fluctuations in the PCV of the birds including the uninfected control birds cannot be readily explained; however, the declines were more severe in the infected birds particularly the broilers than in the uninfected controls and this could be an indication of the pathogenic nature of the parasites. Intestinal haemorrhage and bloody diarrhoea are common occurrences in poultry coccidiosis particularly when* E. tenella* and* E. necatrix* are involved and have been shown to be the main reason for the anaemia.* Eimeria* infections of chickens often lead to disruption of the integrity of intestinal mucosa with resultant reduced appetite and poor digestion and absorption and subsequent poor growth, poor weight gain, or weight loss [5].

The PCV started appreciating towards the end of the study 17(D16) and this coincided with the gradual disappearance of clinical signs especially bloody diarrhoea, although it was still common in the indigenous chickens. Postmortem intestinal haemorrhages were also still more common in the indigenous chickens than in their broiler counterparts, where the healing of haemorrhagic lesions was more advanced, suggesting that the infection was more severe in the indigenous chickens although their PCV was not as low as that of broilers. The pathogenic effect and clinical signs might have been more common in the indigenous chickens due to their small size with the resultant large number of oocysts per kilogramme of body weight when compared with their broiler counterparts. Another potential of the broiler to survive the infection better might have been their greater feed intake with enough nutrients required for tissue repairs and immune response.

Parasitic diseases in general and coccidiosis in particular are known to cause reduced feed intake; however the reduction in consumption in this study was only minimal in the infected birds. This may partially explain why the birds generally gained weight throughout this study.

## 5. Conclusion

It was concluded that the caecal isolate of* Eimeria* oocysts was both infective and pathogenic to the three-week-old broiler and indigenous chickens. It was also observed that the response of the chicken to* Eimeria* infections was dependent on the dose level of the parasite administered to the birds, with both clinical signs and pathogenic effect becoming more severe with increasing dose level. Contrary to what could be expected however, the indigenous chickens showed more severe clinical signs than the broilers which could lead to the suggestion that the indigenous chickens are more susceptible than their broiler counterparts, although the PCV of broilers were much lower than that of the indigenous chickens. None of the two breeds of chickens, however, showed a clear-cut potential to resist the infections more than the other thereby dampening the hope of breeding hybrids of the two breeds which could be both larger than the Nigerian indigenous chicken and more resistant to* Eimeria* infections than the broiler chicken. However, these findings should be further investigated in future studies.

## Figures and Tables

**Table 1:** Experimental design.

Group	Breed	Number of chickens	Dose level	Necropsy (25th day of infection)
A	Broiler chickens	8	2500	Day 25
B	Broiler chickens	8	5000	Day 25
C	Broiler chickens	8	100.000	Day 25
D	Uninfected broiler chickens	8	0	Day 25
E	Local chickens	4	2500	Day 25
F	Local chickens	4	5000	Day 25
G	Local chickens	4	100.000	Day 25
H	Uninfected local chickens	4	0	Day 25
